# A phase 2 trial of long-acting TransCon growth hormone in adult GH deficiency

**DOI:** 10.1530/EC-17-0007

**Published:** 2017-02-14

**Authors:** Charlotte Höybye, Andreas F H Pfeiffer, Diego Ferone, Jens Sandahl Christiansen, David Gilfoyle, Eva Dam Christoffersen, Eva Mortensen, Jonathan A Leff, Michael Beckert

**Affiliations:** 1 Department of EndocrinologyMetabolism and Diabetology, Karolinska University Hospital and Department of Molecular Medicine and Surgery, Karolinska Institute, Stockholm, Sweden; 2 Charité Universitätsmedizin BerlinCampus Benjamin Franklin, Klinik für Endokrinologie & Stoffwechselmedizin, Berlin, Germany; 3 IRCCS AOU San Martino-ISTUniversità di Genova – Endocrinologia DiMI, Dipartimento di Medicina Interna e Specialità Mediche, & CEBR, Centro di Eccellenza per la Ricerca Biomedica, Genova, Italy; 4 Medicinsk Endokrinologist Afd.MEA, NBG, Århus Sygehus, Århus, Denmark; 5 Ascendis Pharma A/SHellerup, Denmark; 6 Ascendis Pharma Inc.Palo Alto, California, USA

**Keywords:** daily growth hormone, adult growth hormone deficiency, long-acting growth hormone, insulin-like growth factor 1

## Abstract

TransCon growth hormone is a sustained-release human growth hormone prodrug under development in which unmodified growth hormone is transiently linked to a carrier molecule. It is intended as an alternative to daily growth hormone in the treatment of growth hormone deficiency. This was a multi-center, randomized, open-label, active-controlled trial designed to compare the safety (including tolerability and immunogenicity), pharmacokinetics and pharmacodynamics of three doses of weekly TransCon GH to daily growth hormone (Omnitrope). Thirty-seven adult males and females diagnosed with adult growth hormone deficiency and stable on growth hormone replacement therapy for at least 3 months were, following a wash-out period, randomized (regardless of their pre-study dose) to one of three TransCon GH doses (0.02, 0.04 and 0.08 mg GH/kg/week) or Omnitrope 0.04 mg GH/kg/week (divided into 7 equal daily doses) for 4 weeks. Main outcomes evaluated were adverse events, immunogenicity and growth hormone and insulin-like growth factor 1 levels. TransCon GH was well tolerated; fatigue and headache were the most frequent drug-related adverse events and reported in all groups. No lipoatrophy or nodule formation was reported. No anti-growth hormone-binding antibodies were detected. TransCon GH demonstrated a linear, dose-dependent increase in growth hormone exposure without accumulation. Growth hormone maximum serum concentration and insulin-like growth factor 1 exposure were similar after TransCon GH or Omnitrope administered at comparable doses. The results suggest that long-acting TransCon GH has a profile similar to daily growth hormone but with a more convenient dosing regimen. These findings support further TransCon GH development.

## Introduction

Hypothalamic–pituitary diseases and/or injury can lead to adult growth hormone deficiency (AGHD) (1). The decrease (or total loss) of growth hormone (GH) production, with a subsequent disruption in the hormone’s relationship with insulin-like growth factor 1 (IGF1), leads to abnormal body composition and metabolism as well as psychological impairment (1, 2). Left untreated, AGHD is associated with decreased bone mineral density, increased risk of cardiovascular mortality as compared to age-matched controls and poor quality of life (3, 4, 5, 6).

Recombinant human GH, also known as somatropin, is produced by inserting cDNA coding for human GH into *Escherichia coli*. It became commercially available in the mid-1980s and is identical to endogenous GH. To date, AGHD standard-of-care treatment consists of daily subcutaneous GH injections. However, a significant portion of patients are poorly adherent to treatment (7), up to 65% in one study, attributed to the inconvenience and discomfort of daily injections, concerns about efficacy and treatment length beyond 2 years (8). Caremark Inc. data showed that prescription refills dropped to 54% within the first 11 months of GH therapy initiation (8). Invariably, poor adherence leads to suboptimal efficacy (6). Given that GH replacement has benefits throughout life (1), many patients will likely need treatment for years. Thus, a safe and efficacious product with less frequent dosing would be a considerable improvement over currently available daily regimens and may lead to increased adherence, with consequent positive physiological effects and better quality of life.

Given that GH receptors are located on virtually every cell in the body and facilitate GH’s many functions, any GH replacement product should be able to reach GH receptors. Currently, a variety of long-acting GH therapies are in development. Most are chemically modified proteins that often extend GH’s half-life by molecular enlargement. However, this approach is potentially problematic if tissue penetration is inadequate. For example, GH crosses the blood–brain barrier to exert its cognitive effects (9) as well as directly stimulates osteoblasts in bone formation (10). If a small molecule becomes too bulky and cannot reach its receptors, efficacy may be compromised (11). Thus, for any long-acting GH to have the multi-faceted effects necessary for successful AGHD treatment, it is important that it mimic endogenous GH as closely as possible in size and other properties.

**Figure 1:**
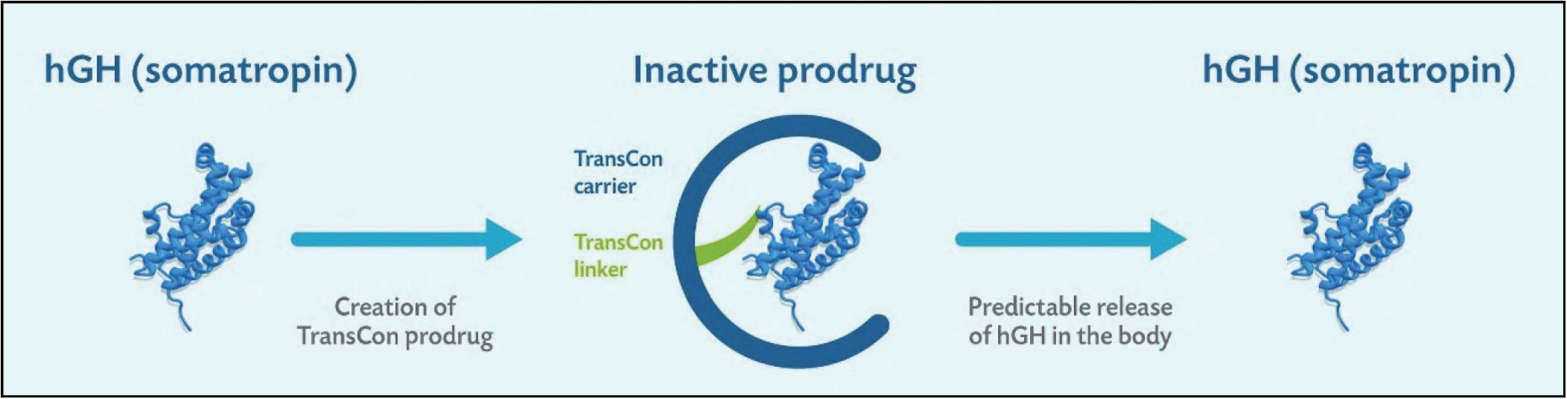
TransCon GH, a sustained-release inactive prodrug consisting of parent drug, unmodified GH, transiently bound to a carrier, methoxypolyethylene glycol (mPEG), via a proprietary linker that is auto-hydrolyzed under physiologic pH and temperature.

TransCon GH is a sustained-release inactive prodrug consisting of a parent drug, unmodified 22 kDa GH equivalent to endogenous GH, transiently bound to a carrier molecule, methoxy polyethylene glycol (mPEG), via a proprietary low-molecular-weight TransCon Linker. The inert mPEG acts as a carrier, extending GH circulation time in the body through a shielding effect that minimizes GH receptor binding and renal excretion, thereby largely inactivating GH until its release (Fig. 1). Over a one-week period, TransCon GH frees fully active GH via auto-hydrolysis of the TransCon linker in a controlled manner based on physiologic pH and temperature. As such, the TransCon technology is designed to maintain the same mode of action and distribution as daily administered GH by allowing the sustained release of unmodified recombinant GH.

The purpose of this investigation was to compare the safety (including tolerability and immunogenicity), pharmacokinetics (PK) and pharmacodynamics (PD) of three different doses of weekly TransCon GH to that of daily recombinant GH in patients with AGHD.

## Materials and methods

### Trial design

This was a Phase 2 multi-center, randomized, open-label, parallel group, active-controlled trial of three different doses of weekly TransCon GH compared to daily Omnitrope. The trial was conducted at 4 reference centers in Sweden, Denmark, Germany and Italy. Institutional review board and independent ethics committee approval as well as signed informed consent from subjects was obtained prior to any trial-specific procedures. The ClinicTrials.gov identifier is Nbib1247675. The EudraCT number is 2010-021523-28.

### Population

Males and females (between the ages of 20 and 70 years with a body mass index (BMI) of 19.0–36.0 kg/m^2^) diagnosed with AGHD (defined according to the Growth Hormone Research Society Consensus Guidelines of 1998 and 2007) (1, 12) who were stable on GH replacement therapy for at least 3 months were enrolled. Fertile females were required to have a negative pregnancy test at the time of enrollment and agree to use contraception during the trial. Subjects were excluded if they had a known history of anti-GH antibodies; were breast-feeding; had a current or (within 5 years) prior malignancy; had uncontrolled diabetes mellitus (defined as an HbA1c over 8.0% and/or receiving insulin treatment) or had other significant comorbidities that would preclude participation in a clinical trial.

### Trial protocol

Subjects attended a screening visit during which their medical history was reviewed and a physical examination (including vital signs, height, and weight) and an electrocardiogram (ECG) were performed. Screening laboratory tests included GH, IGF1, a pregnancy test (for fertile females), hematology, chemistry, coagulation, urinalysis, insulin, HbA1c and lipids.

Subjects then entered a 14- to 21-day wash-out period after cessation of daily GH after which they were randomized (regardless of their pre-study dose) to one of 3 TransCon GH (ACP-001) doses, i.e., 0.02, 0.04 or 0.08 mg GH/kg/week or Omnitrope 0.04 mg GH/kg/week (divided into 7 equal daily doses) for 4 weeks. On Days 1, 8, 15 and 22, TransCon GH-treated subjects were dosed on-site subcutaneously via a syringe with a 30-gauge needle at rotating locations on the abdomen. From Days 1 to 28, Omnitrope-treated subjects self-dosed at home subcutaneously with the 31-gauge Omnitrope Pen at rotating locations on the abdomen and thigh.

### Safety, pharmacokinetics, and pharmacodynamics assessments

Safety assessments, made prior to dosing at select visits as well as at the safety follow-up visit (Day 42), included vital signs with weight, physical examination, ECG, immunogenicity evaluation and clinical laboratory testing. Serum for anti-GH-binding antibodies was drawn at screening and Days 0, 8, 22, 29 and 42 and analyzed using a validated ELISA (Millipore BioPharma Services, currently known as Eurofins Pharma Bioanalysis Services UK Limited). If samples were confirmed anti-GH binding antibody positive, samples were subsequently analyzed for the presence of neutralizing antibodies using a validated cell-based proliferation assay. Clinical laboratory safety tests, including hematology, chemistry, coagulation, urinalysis, insulin, HbA1c and lipids were drawn on Days 0, 2, 8, 15, 22, 29 and 42. Results in standard international units were converted using the Society for Biomedical Diabetes Research SI Unit Conversion Calculator (http://www.soc-bdr.org/rds/authors/unit_tables_conversions_and_genetic_dictionaries/conversion_in_si_units/index_en.html). Subjects were monitored for adverse events (AEs) throughout the study. Local tolerability was evaluated by injection site reactions (defined as erythema, swelling and pain) at 8, 12 and 24 h after dose on Days 8 and 15 as well as by patient diary.

Blood samples for PK and PD assessments in the TransCon GH cohorts were collected at Day 0 and pre-dose on Days 1, 8, 15 and 22. After the first (Day 1) and fourth (Day 22) doses, post-dose samples were collected repeatedly for up to seven (Day 8) and twenty (Day 42) days, respectively. Additional samples were collected on Days 9 and 21. In Omnitrope-treated subjects, samples were collected at pre-dose on Day 1 and then repeatedly for up to 24 h after dose administration on Days 1 and 22. Additional samples were collected pre-dose on 14 occasions (Days 3–28) and post-dose on Days 29, 31, 35 and 42.

Analyses of GH and IGF1 were performed by Celerion Inc. (Lincoln, NE, USA). GH was quantified in serum by a validated commercially available sandwich ELISA (ALPCO Diagnostics, Salem, NH, USA), whereas plasma IGF1, the primary PD biomarker, was quantified by a validated commercially available sandwich ELISA (R&D Systems) using WHO International Standard 02/254 as reference material.

Baseline GH was defined as the last available measurement prior to the first dose (usually Day 1) and set to zero as all baseline samples were below the quantification limit. Maximum GH concentration (*C*_max_) was defined as the highest serum concentration achieved post-dose following Days 1 and 22 administrations for all cohorts. Area under the curve (AUC) for TransCon GH-treated subjects was calculated based on drug concentration at time 0–168 h after-dose on Days 1 and 22 using the linear trapezoidal rule for the ascending part of the concentration–time curve and the logarithmic trapezoidal rule for the descending part. AUC for Omnitrope-treated subjects was calculated based on drug concentration at time 0–24 h post-dose on Days 1 and 22 multiplied by 7 to be comparable to weekly TransCon GH. For subjects with fewer than three consecutive data points above the lower limit of quantification (LLOQ), AUC was not calculated, and these subjects were excluded from the overall mean AUC calculation.

For PD, baseline IGF1 was defined as the last available measurement prior to the first dose (usually Day 1). Time to maximum efficacy (*T*_Emax_) was defined as the time needed to attain the maximum IGF1 effect (*E*_max_). The IGF1 area under the effect curve (AUEC) was calculated based on the difference of AUEC areas above and below zero (ie, AUEC = AUEC_above_ − AUEC_below_) for both TransCon GH and Omnitrope-treated subjects using the same methodology as for PK.

### Statistical analysis

Demographics, vital signs including height and weight and ECG as well as GH, IGF1 and anti-GH antibody data were analyzed by descriptive statistics using SAS, version 9 or higher. AEs were summarized but not statistically evaluated. Demographics, baseline characteristics, safety and tolerability were analyzed in all subjects who were randomized and received at least one dose of test product with an analysis of variance performed for age, weight and height. PK and PD parameters were assessed for subjects who received at least one dose of test product, had at least one primary variable measurement drawn and attended all visits up to and including Day 28; PK and PD analysis were performed using WinNonLin, version 5.2.

## Results

Thirty-seven subjects satisfied inclusion and exclusion criteria and were randomized into four cohorts. Cohort 1 (*n* = 10) received TransCon GH 0.02 mg/kg/week, Cohort 2 (*n* = 10) received TransCon GH 0.04 mg/kg/week, Cohort 3 (*n* = 9) received TransCon GH 0.08 mg/kg/week and Cohort 4 (*n* = 8) received Omnitrope 0.04 mg/kg/week (in 7 divided doses). All 37 subjects were included in demographic and baseline characteristic analyses (Table 1). The majority of patients (28/37; 76%) had multiple pituitary hormone deficiencies, i.e., one or more hypothalamic–pituitary axes deficiencies in addition to AGHD.

**Table 1:** Demographic and baseline characteristics of TransCon GH and Omnitrope cohorts.

	**Cohort 1 TransCon GH 0.02 mg/kg/week**	**Cohort 2 TransCon GH 0.04 mg/kg/week**	**Cohort 3 TransCon GH 0.08 mg/kg/week**	**Cohort 4 Omnitrope 0.04 mg/kg/week**
At screening	*n* = 10	*n* = 10	*n* = 9	*n* = 8
Male, female	5, 5	4, 6	4, 5	5, 3
Mean age (years) (s.d.)	55.7 (12.6)	45.9 (15.0)	51.6 (18.1)	44.0 (15.7)
Mean weight (kg) (s.d.)	82.3 (19.8)	79.6 (17.0)	92.5 (20.5)	74.7 (16.6)
Mean height (cm) (s.d.)	171.7 (10.9)	168.5 (11.4)	172.7 (10.0)	170.1 (12.9)
Mean BMI (kg/m^2^) (s.d.)	27.6 (4.4)	27.9 (4.4)	30.7 (4.4)	25.7 (4.4)
At Day 0	*n* = 10	*n* = 8	*n* = 9	*n* = 8
Mean IGF-1 (ng/mL) (s.d.)	41.9 (16.8)	42.8 (26.9)	42.6 (12.7)	54.0 (25.6)

Four subjects withdrew from the study, two each from Cohorts 1 and 2. In Cohort 1, one subject withdrew due to comorbidities; the other withdrew for personal reasons; both were, however, included in the PK and PD analyses. In Cohort 2, each subject withdrew due to adverse events with their withdrawal occurring before Day 28; both were therefore excluded from the PK and PD analyses, leaving 35 subjects. Further details about withdrawal due to adverse events may be found under ‘Safety’ section.

The cohorts were balanced with respect to gender; 18 (49%) were male, 19 (51%) were female. All were Caucasian except two who were Arabic and non-Caucasian Hispanic, respectively. Mean age was 49.5 years, mean BMI was 28 kg/m^2^ and mean IGF1 at baseline after wash-out was 45.0 ng/mL.

### Safety

A total of 178 AEs were reported; 78.4% (29/37) of subjects experienced at least 1 AE and 51.4% (19/37) experienced at least 1 AE definitely, probably or possibly related to the study medication. Fatigue and headache were reported in all groups and were the most frequent drug-related AEs observed (Table 2).

**Table 2:** Treatment-emergent adverse events occurring in >1 subject in any cohort.

	**Cohort 1 TransCon GH 0.02 mg/kg/week** ***n* = 10** (%)	**Cohort 2 TransCon GH 0.04 mg/kg/week** ***n* = 10** (%)	**Cohort 3 TransCon GH 0.08 mg/kg/week** ***n* = 9** (%)	**Cohort 4 Omnitrope 0.04 mg/kg/week** ***n* = 8** (%)
Fatigue	3 (30)	3 (30)	1 (11)	2 (25)
Headache	2 (20)	3 (30)	2 (22)	2 (25)
Oropharyngeal pain	1 (10)	3 (30)	2 (22)	2 (25)
Nausea	0 (0)	3 (30)	2 (22)	1 (13)
Nasopharyngitis	0 (0)	1 (10)	2 (22)	2 (25)
Diarrhea	1 (10)	2 (20)	0 (0)	1 (13)
Arthralgia	2 (20)	1 (10)	1 (11)	0 (0)
Application site erythema	2 (20)	0 (0)	0 (0)	1 (13)
Application site induration	2 (20)	0 (0)	0 (0)	1 (13)
Cough	0 (0)	3 (30)	0 (0)	0 (0)
Back pain	1 (10)	0 (0)	2 (22)	0 (0)
Asthenia	2 (20)	0 (0)	0 (0)	0 (0)
Influenza-like illness	0 (0)	2 (20)	0 (0)	0 (0)
Affect lability	2 (20)	0 (0)	0 (0)	0 (0)
Depressed mood	2 (20)	0 (0)	0 (0)	0 (0)
Insomnia	2 (20)	0 (0)	0 (0)	0 (0)
Stress	0 (0)	0 (0)	0 (0)	2 (25)
Hematuria	0 (0)	0 (0)	0 (0)	2 (25)

Treatment-related AE incidence was similar in TransCon GH Cohorts 1 and 3 and Omnitrope Cohort 4 (40, 44, and 50%, respectively) compared to 70% in the TransCon GH Cohort 2 (Table 3). No AEs led to death. Two serious adverse events (SAEs) occurred in one subject, a 70-year-old female with a history of multiple adrenal crises in Cohort 1 receiving TransCon GH 0.02 mg/kg/week. Six days after treatment initiation, she again experienced a severe adrenal crisis requiring hospitalization. After recovery, she experienced a second SAE, moderate pleuritic chest pain likely caused by a pulmonary infiltrate. Although neither SAE was considered related to TransCon GH, the patient was withdrawn from the trial 7 days after the fourth injection.

**Table 3:** Summary of adverse events by cohort.

	**Cohort 1 TransCon GH 0.02 mg/kg/week *n* = 10** (%)	**Cohort 2 TransCon GH 0.04 mg/kg/week *n* = 10** (%)	**Cohort 3 TransCon GH 0.08 mg/kg/week *n* = 9** (%)	**Cohort 4 Omnitrope 0.04 mg/kg/week *n* = 8** (%)
Reported	6 (60)	9 (90)	7 (78)	7 (88)
Serious	1 (10)	0 (0)	0 (0)	0 (0)
Treatment related	4 (40)	7 (70)	4 (44)	4 (50)
Leading to withdrawal	1 (10)	2 (20)	0 (0)	0 (0)

Two additional subjects withdrew because of AEs. One subject in Cohort 2 receiving TransCon GH 0.04 mg/kg/week developed mild diarrhea one day after treatment initiation, considered possibly related to the study drug. A second subject receiving the same TransCon GH dose experienced a mild influenza-like illness two days after treatment initiation, considered unlikely to be related to the study drug.

No lipoatrophy or nodule formation at the injection site was reported. Nine subjects experienced at least one injection site reaction; there was no clear difference in these reactions between TransCon GH and Omnitrope-treated subjects. Eight subjects (2 in Cohort 1 and 3, respectively; 3 in Cohort 2 and 1 in Cohort 4) experienced injection site erythema. One subject in both Cohorts 1 and 2 experienced injection site pain, whereas 1 subject in Cohort 1 experienced injection site swelling.

One subject treated with GH since 2007 was found to possess pre-existing non-neutralizing anti-GH-binding antibodies. Of the remaining 36 subjects, anti-GH-binding antibodies were not detected.

The mean BMI of TransCon GH Cohorts 1–3 was 28.8 kg/m^2^ at screening and 29.0 kg/m^2^ at Day 29 compared to 25.7 kg/m^2^ and 25.8 kg/m^2^ for Omnitrope Cohort 4, respectively (Table 4). Of the 19 subjects experiencing treatment-related AEs, 7 of 15 TransCon GH vs 1 of 4 Omnitrope-treated subjects had a BMI ≥30.0. The mean total cholesterol of TransCon GH Cohorts 1–3 decreased from 213 on Day 0 to 203 mg/dL on Day 29 compared to a decrease from 208 to 187 mg/dL in Omnitrope Cohort 4, respectively. For both TransCon GH Cohorts 1–3 and Omnitrope Cohort 4, HbA1c was 5.7% at Day 0 and 5.6% at Day 29.

**Table 4:** Summary of clinical safety parameters by cohort.

		**Cohort 1 TransCon GH 0.02 mg/kg/week**	**Cohort 2 TransCon GH 0.04 mg/kg/week**	**Cohort 3 TransCon GH 0.08 mg/kg/week**	**Cohort 4 Omnitrope 0.04 mg/kg/week**
Mean BMI (kg/m^2^) (s.d.)		(*n* = 9)	(*n* = 8)	(*n* = 9)	(*n* = 8)
	Screening	27.5 (4.7)	28.2 (3.8)	30.7 (4.4)	25.7 (4.4)
	Day 29	27.6 (4.6)	28.4 (4.0)	30.9 (4.4)	25.8 (4.7)
Mean total cholesterol (mg/dL) (s.d.)		(*n* = 8)	(*n* = 8)	(*n* = 9)	(*n* = 8)
	Day 0	228 (63)	203 (48)	209 (33)	208 (47)
	Day 29	221 (50)	194 (41)	194 (25)	187 (31)
Mean HDL cholesterol (mg/dL) (s.d.)		(*n* = 8)	(*n* = 8)	(*n* = 9)	(*n* = 8)
	Day 0	67 (27)	54 (17)	57 (13)	64 (26)
	Day 29	63 (26)	51 (18)	55 (14)	58 (23)
Mean LDL cholesterol (mg/dL) (s.d.)		(*n* = 8)	(*n* = 8)	(*n* = 9)	(*n* = 8)
	Day 0	138 (53)	126 (26)	127 (34)	122 (40)
	Day 29	132 (41)	120 (30)	113 (26)	109 (27)
Mean triglycerides (mg/dL) (s.d.)		(*n* = 8)	(*n* = 8)	(*n* = 9)	(*n* = 8)
	Day 0	124 (55)	122 (74)	145 (103)	97 (68)
	Day 29	140 (71)	123 (76)	129 (47)	100 (103)
Mean glucose (mg/dL) (s.d.)		(*n* = 8)	(*n* = 7)	(*n* = 9)	(*n* = 7)
	Day 0	77 (13)	89 (23)	74 (19)	93 (30)
	Day 29	80 (12)	84 (20)	78 (19)	85 (14)
Mean HbA1c (%) (s.d.)		(*n* = 3)	(*n* = 3)	(*n* = 4)	(*n* = 2)
	Day 0	5.9 (0.2)	5.6 (0.5)	5.7 (0.5)	5.7 (NA)
	Day 29	5.8 (0.2)	5.5 (0.3)	5.6 (0.4)	5.6 (NA)

NA, not applicable.

### Pharmacokinetics

**Figure 2:**
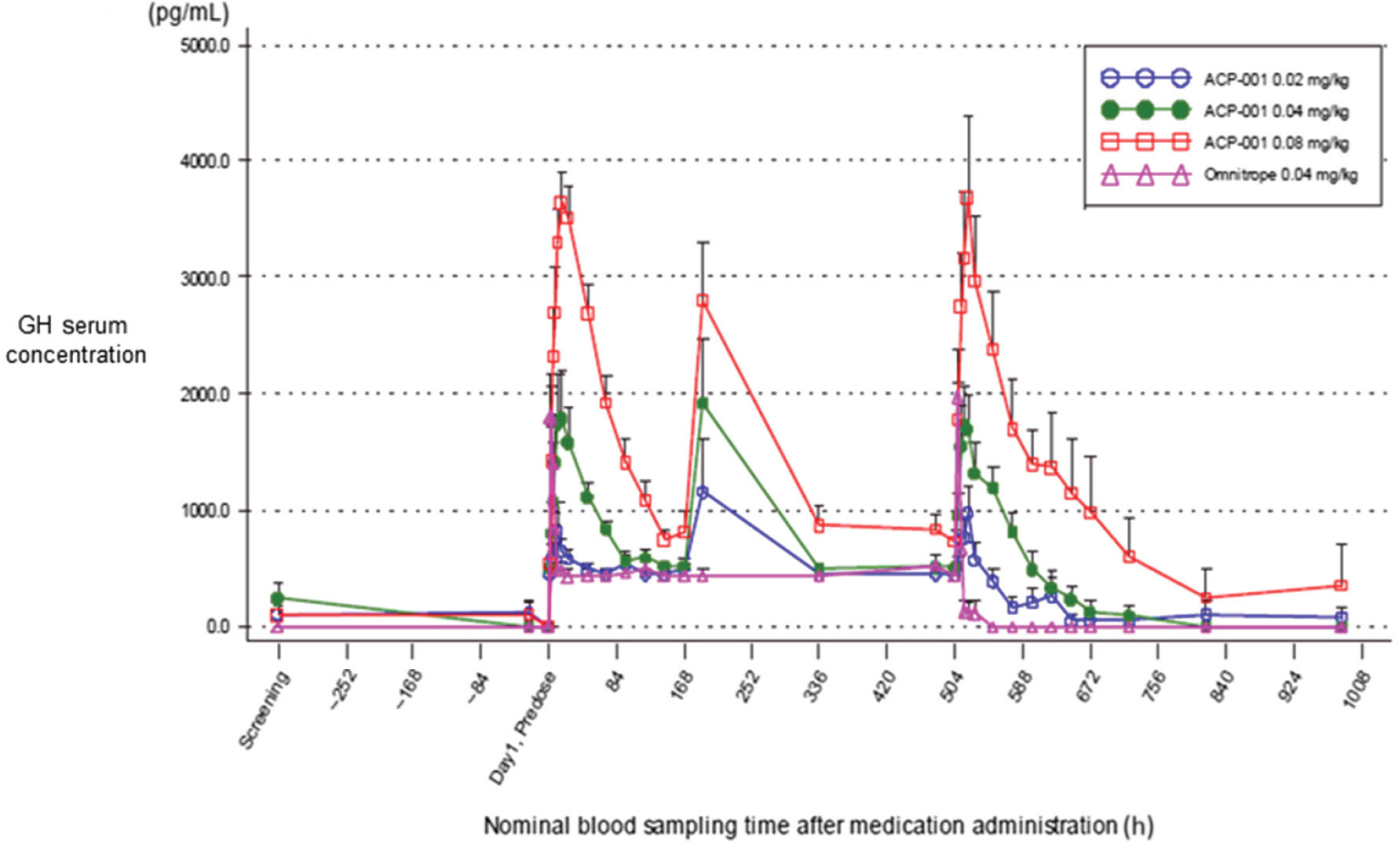
GH serum concentration (pg/mL), arithmetic means (+s.d.), linear scale, untransformed data, following weekly administration of TransCon GH or daily Omnitrope from screening to day 42. Omnitrope-treated subjects sampled intensively for 24 h after doses on Days 1 (0 h) and 22 (504 h). All other time points between Day 3 (48 h) to Day 21 (480 h) and Day 24 (552 h) to Day 42 (948 h) were sampled prior to dosing. Data from two time points from one Cohort 3 subject were excluded due to sample mishandling with subsequent outlier results.

The GH serum concentration profiles after subcutaneous administration of TransCon GH from Days 1 to 42 are presented in Fig. 2. TransCon GH released GH in a sustained manner over the intended 168-h dose interval. A linear, dose-dependent increase in GH exposure (*C*_max_ and AUC_0–168 h_) was observed, without accumulation, after multiple administrations of TransCon GH. As expected, GH median *T*_max_ was reached more slowly than that of Omnitrope administration, ranging from 14–16 h compared to 2–4 h for Omnitrope. GH peak exposures (*C*_max_) after administration of TransCon GH or Omnitrope at comparable doses (0.04 mg/kg/week) were similar at both Weeks 1 and 4. The PK summary for Weeks 1 and 4 is presented in Table 5. As the observed GH PK profile reflects TransCon GH linker hydrolysis kinetics, TransCon GH and GH PK show similar overall profiles, dose dependency and lack of accumulation (data not shown).

**Table 5:** Summary of growth hormone PK for all 4 cohorts (Weeks 1 and 4; untransformed data).^a^

	Cohort 1 TransCon GH 0.02 mg/kg/week		Cohort 2 TransCon GH 0.04 mg/kg/week		**Cohort 3 TransCon GH 0.08 mg/kg/week**		**Cohort 4 Omnitrope 0.04 mg/kg/week**	
Week	1	4	1	4	1	4	1	4
Median *T*_max_ (h)	(*n* = 8)	(*n* = 9)	(*n* = 8)	(*n* = 8)	(*n* = 9)	(*n* = 9)	(*n* = 8)	(*n* = 7)
	14.0	1.0	16.0	14.1	16.0	16.0	2.1	4.0
Mean *C*_max_ (ng/mL) (s.d.)	(*n* = 10)	(*n* = 10)	(*n* = 8)	(*n* = 8)	(*n* = 9)	(*n* = 9)	(*n* = 8)	(*n* = 8)
	0.8 (0.8)	1.2 (0.8)	1.9 (1.1)	1.9 (0.9)	4.0 (0.8)	3.8 (2.0)	2.1 (0.9)	2.0 (1.1)
Mean AUC (h * ng/mL) (s.d.)	(*n* = 4)	(*n* = 4)	(*n* = 8)	(*n* = 7)	(*n* = 9)	(*n* = 9)	(*n* = 7)	(*n* = 2)
	86 (31)	89 (22)	133 (39)	154 (32)	302 (94)	318 (197)	132 (25)	146 (NA)
Mean accumulation, AUC^b^		(*n* = 3)		(*n* = 7)		(*n* = 9)		(*n* = 2)
	NA	1.4	NA	1.2	NA	1.0	NA	1.1

*C*_max_ and AUC values are reported as arithmetic means (s.d.).

a Excludes data from 2 mishandled samples from one subject. ^b^Mean accumulation ratio between AUC at Week 4 compared to Week 1 based on TransCon GH 0–168 h; Omnitrope (0–24 h) * 7.

NA, not applicable.

Of note, one subject in TransCon GH Cohort 3 had very high GH levels at 4 and 6 h after the first injection, confirmed by sample reanalysis. The remainder of this subject’s GH profile was comparable to others in the cohort. As such, the spike was attributed to sample mishandling, and the two data points were excluded from the PK analysis.

### Pharmacodynamics

**Figure 3:**
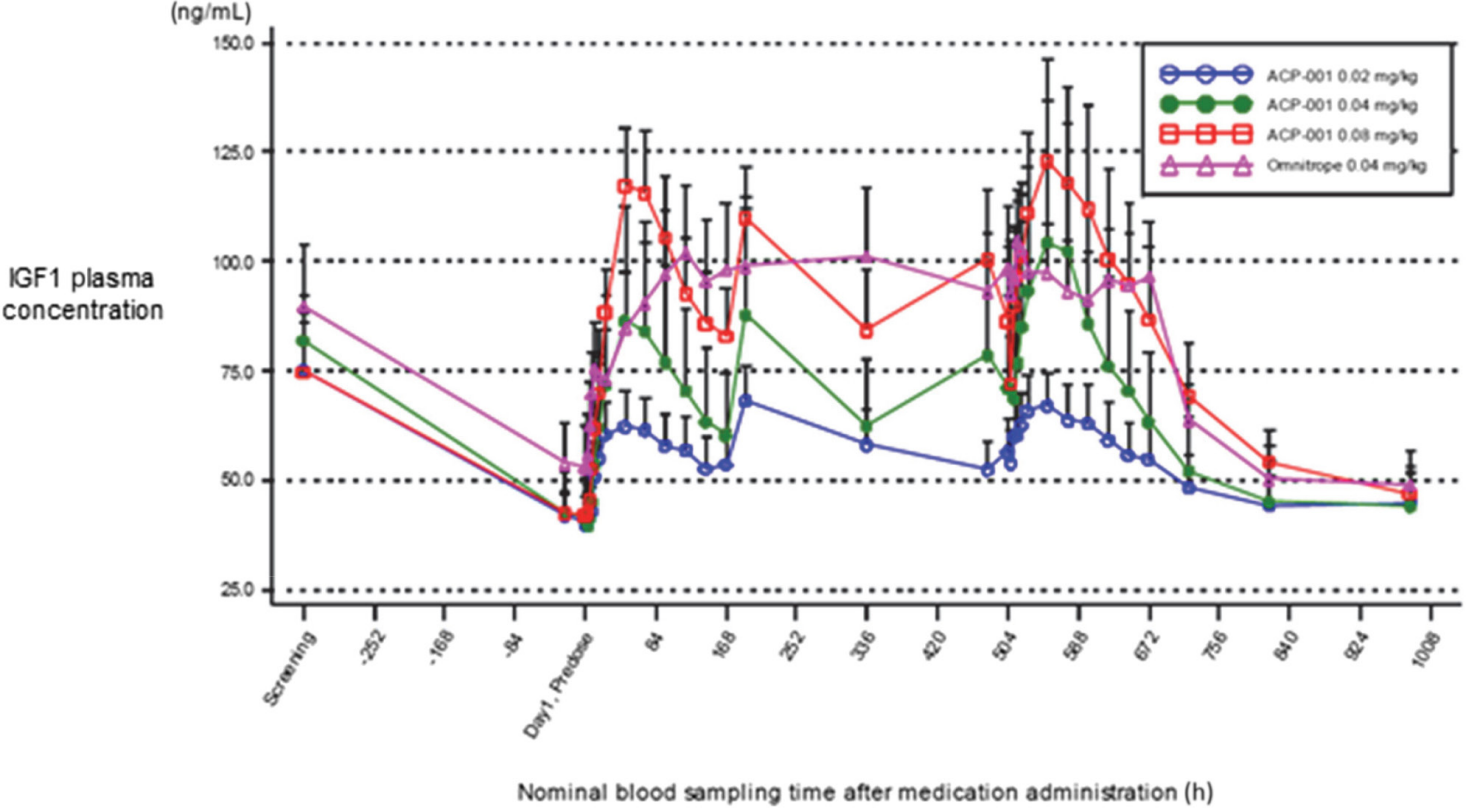
Plasma IGF1 response (ng/mL) to weekly TransCon GH or daily Omnitrope from Day 1 to 42, untransformed arithmetic mean (+s.d.).

Plasma IGF1 levels increased above baseline at all doses of TransCon GH and Omnitrope (Fig. 3). After repeat dosing of TransCon GH and Omnitrope, IGF1 trough levels remained above baseline. Maximum observed response (*E*_max_) was higher after the fourth dose compared to the first dose, consistent with multiple GH doses required to establish a stable IGF1 response. Baseline corrected IGF1 exposure (*E*_max_ and AUEC_0–168 h_) after the first and fourth doses was essentially linear across TransCon GH doses. IGF1 exposure after administration of TransCon GH (AUEC_0–168 h_) or Omnitrope (AUEC_0–24 h_ × 7) 0.04 mg/kg/week was similar. The PD summary at steady state is presented in Table 6.

**Table 6:** Summary of IGF1 PD for all 4 cohorts (Week 4; untransformed data).

	**Cohort 1 TransCon GH 0.02 mg/kg/week**	**Cohort 2 TransCon GH 0.04 mg/kg/week**	**Cohort 3 TransCon GH 0.08 mg/kg/week**	**Cohort 4 Omnitrope 0.04 mg/kg/week**
Median *T*_Emax_^a^ (h)	(*n* = 10)	(*n* = 8)	(*n* = 9)	(*n* = 8)
	24.0	60.0	48.0	12.0
Mean *E*_max_^a^ (ng/mL) (s.d.)	(*n* = 10)	(*n* = 8)	(*n* = 9)	(*n* = 8)
	71.8 (26.9)	108.6 (91.7)	125.6 (70.1)	109.8 (37.1)
AUEC^b^ (h * ng/mL) (s.d.)	(*n* = 8)	(*n* = 8)	(*n* = 9)	(*n* = 8)
	10,441 (4560)	14,314 (11,432)	17,851 (10,099)	16,656 (5624)

*E*_max_ and AUEC are reported as arithmetic means (s.d.).

a TransCon GH 0–168 h; Omnitrope 0–24 h. ^b^TransCon GH 0–168 h; Omnitrope (0–24 h) * 7.

## Discussion

Overall, TransCon GH was well tolerated; the two SAEs in one patient were not considered treatment related. In contrast to several other weekly GH products based on protein enlargement (13, 14, 15), no lipoatrophy or nodule formation occurred at injection sites in this trial. And no treatment-emergent anti-GH antibodies were detected. Clinical safety parameters were stable with similar directional trends between TransCon GH and Omnitrope. After repeat doses, TransCon GH demonstrated a linear, dose-dependent increase in GH peak exposure without accumulation and a similar *C*_max_ as compared to Omnitrope at equivalent weekly dosing. IGF1 exposure after equivalent dosing of TransCon GH and Omnitrope was also similar.

AGHD is associated with increased mortality, mainly due to cardiovascular risk. GH deficiency contributes to visceral obesity, leading to insulin resistance and dyslipidemia (especially high LDL), diabetes mellitus and chronic inflammation (specifically increased C-reactive protein). The net result is increased atherosclerosis and reduced cardiac function with a subsequent increased risk of cardiovascular events and mortality (16). In a study by Hoffman and coworkers, 166 AGHD subjects were treated with daily GH. After 12 months, they had significantly decreased total body fat, increased lean body mass and improvements in their total cholesterol and LDL (17). In their review of AGHD-randomized trial participants, Maison and coworkers also found that GH treatment is associated with a significant positive effect on cardiac function, attributed to the trophic effects of GH (18).

The current study was a Phase 2 PK, PD and dose-finding study, making definitive conclusions about the clinical safety parameters from a long-term perspective premature. However, TransCon GH mimicked the directional trend of similarly dosed Omnitrope across BMI, lipids and HbA1c. Given TransCon GH’s comparable results to Omnitrope with regard to GH serum concentration and the fact that the active drug in both cases is unmodified human GH, similar improvement in overall cardiovascular morbidity and mortality as compared to daily GH treatment might be expected in patients with AGHD who receive TransCon GH long term. This will need to be confirmed in future studies.

Daily GH therapy is supported by many years of safety data (19); GH replacement in AGHD is well tolerated, and there is no evidence of increased risk of de novo or recurrent malignancy (6). IGF1 is the best and most used serum marker for GH dose titration (1). From a safety perspective, it is therefore encouraging that TransCon GH-mediated IGF1 levels were similar to those seen for Omnitrope after administration of equivalent doses over 4 weeks.

One subject, with a history of multiple prior adrenal crises, did suffer an adrenal crisis in this study. The investigator did not attribute it to TransCon GH. However, daily GH therapy may render recipients with low adrenal reserve hypoadrenal (20). Further study is needed to determine if daily and long-acting GH therapies share similar adrenal risks.

Unlike pediatric GH deficiency where dosing is based by weight, AGHD dosing is more complex and individualized, taking into account age, gender and IGF1 levels (6). Upward (or downward) titration is required to maximize benefits and minimize side effects all while targeting normal age and gender IGF1 levels (21). It is worth noting that subjects in this trial were randomly assigned to fixed test drug dose levels irrespective of their pre-study GH dose, which may have been higher (or lower). Some AEs could therefore be anticipated as subjects were not necessarily placed on their ideal dose. That fatigue and headache were subsequently the most common AEs is not surprising given that they are considered typical of both GHD and GH treatment (22).

The expected low titer, non-neutralizing antibody development rate in children receiving Genotropin is approximately 5–12%; adults develop antibodies much less frequently than children (23, 24). One subject was found to have pre-existing non-neutralizing antibodies, likely due to long-term daily GH treatment, the levels of which did not change after dose. However, the remaining subjects did not develop anti-GH-binding antibodies.

This study had certain limitations. An approved long-acting GH product for AGHD with similar safety, efficacy, tolerability and immunogenicity as Omnitrope was not available. Thus, this study is a comparison of two products with different dosing regimens, short-acting versus long-acting GH. These different administration frequencies also made blinding unfeasible. The sample size was small with only 29 subjects receiving TransCon GH. However, AGHD is estimated to affect only 1 per 100,000 annually (6). The main efficacy endpoint was limited to IGF1 levels and thus did not require a large study. Due to sensitivity limitations of the GH assay, several subjects did not have concentration data supporting AUC calculations and were excluded. Mean GH AUC was therefore biased on the high side, particularly in Weeks 1 and 4 of TransCon GH Cohort 1 and Week 4 of Omnitrope Cohort 4. Still, comparable Week 1 AUCs were observed between equivalent doses of TransCon GH Cohort 2 and Omnitrope Cohort 4 supporting similarity in GH exposure.

Overall, weekly TransCon GH, with a mg to mg conversion to commercially available daily GH products, was found to be similar to daily Omnitrope at a GH equivalent dose in terms of adverse events, tolerability, immunogenicity, serum GH and plasma IGF1 levels. The results support efficacy trials in AGHD that will help evaluate TransCon GH’s long-term effects on body composition, bone metabolism and quality of life.

## Declaration of interest

C H: Investigator for Novo Nordisk, Teva, Ascendis Pharma A/S, Versatis, and Handok-Genexine and has consulted for Novo Nordisk and Teva.

## Funding

Funding for this trial was provided by Ascendis Pharma A/S.

## Acknowledgments

This paper is dedicated to the memory of Prof. Jens Sandahl Christiansen, who passed away prior to publication. Jens Sandahl Christiansen was actively involved in all parts of the study, and as the coordinating investigator, he was instrumental in seeing this study to completion. Jens Sandahl Christiansen will be remembered for his long and strong dedication to the field of growth hormone deficiency. The authors would like to thank the subjects, the principal investigators and Christine Andersen and David B Karpf for manuscript preparation assistance.

## Principal investigators

The following individuals were principal investigators at sites enrolling subjects: Jens Sandahl Christiansen (Århus, Denmark), Andreas F H Pfeiffer (Berlin, Germany), Diego Ferone (Geneva, Italy), Charlotte Höybye (Stockholm, Sweden).
